# Predictive factors of Black American emerging adults’ psychological flourishing: a random forest analysis

**DOI:** 10.1038/s41598-026-46864-5

**Published:** 2026-04-17

**Authors:** Nema Kebbeh, Elizabeth Jelsma

**Affiliations:** Department of Psychological, Health, and Learning Sciences, University of Houston, Houston, USA

**Keywords:** Flourishing, Black emerging adults, John Henryism active coping, Racial discrimination, Random forest, SHAP, Health care, Psychology, Psychology

## Abstract

Flourishing reflects positive psychosocial functioning and well-being. In this study, recent psychological distress was indexed using the CDC Healthy Days mental health item (mentally unhealthy days), reverse-coded as good mental health days. Emerging adulthood (18–29) is pivotal for mental health, yet robust predictors of flourishing among Black American emerging adults are understudied. We analyzed data from 513 participants (Mage = 23.6, SD = 3.35) using a 1,000-tree Random Forest with SHapley Additive exPlanations (SHAP). Performance was stable using out-of-bag (OOB) internal validation (OOB R^2^ = 0.267; test R^2^ = 0.245; RMSE = 1.044). JHAC was the dominant predictor, followed by good mental health days and good physical health days; discrimination and sleep showed smaller contributions, and substance use contributed least. Models included demographic covariates (age, education, religion, marital status, employment, female, parenting status, and having children). SHAP interaction values suggested conditional effects involving JHAC, including a pattern consistent with buffering in the association between current discrimination and flourishing. Methodologically, random forest and SHAP yielded transparent, individual-level explanations, revealing nonlinearities and interactions without pre-specification.

**Supplementary Information:**

The online version contains supplementary material available at 10.1038/s41598-026-46864-5.

## Introduction

Flourishing is a broad form of well-being: feeling good and functioning well in daily life, not just the absence of mental illness^1^. Although flourishing is often inversely related to psychological distress, well-being and distress are not simple opposites and are commonly measured separately in population research^2^. It reflects both positive feelings and satisfaction (hedonic aspects) and a sense of meaning, purpose, and social functioning (eudaimonic elements) within a single, broader indicator of mental health^3^. Modern definitions highlight a sense of purpose, meaningful relationships, prosocial engagement, and progress toward personal goals^1^. Because flourishing emphasizes strengths and adaptive functioning across life domains, it complements symptom-focused measures and can inform interventions that aim to help people thrive^4^.

Ages 18–29 are a pivotal period marked by identity exploration, changing roles, and shifting social supports^5^. These years offer opportunities for growth but also bring instability as young adults navigate education, work, relationships, and independent living^6^. Cognitive and psychosocial development continue into the mid-twenties, underscoring the need for supportive contexts and effective coping to foster positive outcomes^6^. Many psychiatric conditions first appear during early adulthood, making this a critical window for prevention and for promoting positive mental health outcomes such as flourishing^7^. Assessing flourishing during this transition can reveal vulnerabilities and strengths that shape long-term well-being^8,9^.

Black American young adults often face stressors such as interpersonal and institutional racial discrimination, vicarious exposure to racialized events, and systemic inequities that increase chronic stress and threaten well-being^10^. Discrimination is consistently linked to poorer mental health, including more symptoms of depression and anxiety and lower life satisfaction^11^. At the same time, culturally grounded strengths such as a positive ethnic–racial identity, family and community support, faith and religious involvement, and collective coping can protect and promote flourishing despite adversity^10,11^. Identifying predictors of flourishing and how these strengths buffer risk is essential for designing culturally responsive interventions that advance equity in positive mental health outcomes^10,11^.

### Predictors of flourishing

Racial discrimination, defined as unfair, differential treatment based on race, is a common experience for people from marginalized groups young people in the United States^12^. Black American emerging adults’ racial discrimination experiences have been shown to increase risk for worse psychological well-being^13^. However, many Black American emerging adults who encounter discrimination do not experience psychological dysfunction, and these varied outcomes may be attributable to differences in coping.

Because of racism, many Black Americans often feel compelled to exert greater effort and determination than their white counterparts to achieve success^14^. One prominent race-relevant coping strategy observed among Black Americans is John Henryism Active Coping (JHAC), named after the folklore figure John Henry, a Black steel driver who famously outperformed a steam-powered drill but died from exhaustion shortly after his victory. This coping style is characterized by traits such as mental vigor, a strong work ethic, and unwavering determination to succeed^15,16^. A substantial body of research has demonstrated that reliance on this high-effort coping approach in response to stress is associated with increased risks of hypertension and other adverse health outcomes^15,17^. Feeling more in control of one’s situation can reduce perceptions of stress^18,19^, which might explain why, among the limited research on high-effort coping and mental health, there is a pattern of higher John Henryism being related to better mental health^14,20–22^. Approaching challenges proactively and with a sense of control supports identity development, a core element of flourishing^23^. Little is still known about the utility of JHAC to protect mental health and promote psychological flourishing among Black American emerging adults faced with racial discrimination, emphasizing that emerging adulthood is a particularly sensitive period for experiencing race-based social rejection^13^, self-confidence and identity development^24^, and low flourishing^25^.

Higher psychological distress is consistently associated with lower well-being and reduced positive functioning^26^. Among Black American young adults, prevalence is high, and mental health services are underused relative to white peers, which can worsen outcomes^27^. Sleep and physical health are also foundational to flourishing in emerging adulthood, with sleep quality a particularly strong predictor^28^. This is a bidirectional process, as people who are flourishing are more likely to rate their general health as excellent^29,30^. In addition to diagnosable conditions, the daily burden of mental health affects how young adults function and find meaning in their lives. Physical health also affects daily role performance, energy levels, and social participation, which may indirectly promote or undermine flourishing in young adults^31^. Sleep represents a fundamental biobehavioral pathway that facilitates emotion regulation, cognitive functioning, and stress recovery, and may contribute to flourishing both directly and through its interconnections with mental and physical health^32^.

Substance use is another barrier to flourishing for Black American young adults^26^. Engaging in fewer risk behaviors, including lower substance use, is characteristic of flourishing, whereas greater involvement signals floundering^26^. For many, however, substance use can function as a maladaptive response to racial stress^33^. Discrimination is positively associated with current cannabis and tobacco use, with psychological distress partly explaining this link; these effects can be especially pronounced among Black American young men who attribute unfair treatment to racism^34^.

### Limitations of linear models and a machine learning alternative

Linear regression is widely used in psychology, but it often struggles to capture the curved relationships and interactions that are common in constructs like flourishing^35,36^. Its core assumptions, including straight-line relationships, constant variance, normal residuals, and independent observations, are frequently violated in psychological data. These violations can bias estimates, increase false positives, and weaken conclusions^37,38^. Linear models also require researchers to specify interaction terms in advance and to choose exact functional forms. This raises the risk of missing essential interactions or fitting the wrong form, which complicates interpretation and can lead to inaccurate inferences^39,40^.

Common features of psychological datasets add further challenges for linear regression. Measures often include error, distributions are not always normal, and data can be clustered or repeated over time^38,41^. Measurement error is especially problematic because it can bias interaction estimates and widen confidence intervals, obscuring actual effects^41,42^. Interaction effects are often minor, context-dependent, and difficult to detect or replicate reliably with traditional linear methods^41,43^. As a result, linear models can underrepresent the complexity of psychological phenomena and may not reflect real-world dynamics well.

Machine learning approaches offer an alternative. Ensemble methods such as Random Forest can model curved relationships and higher-order interactions without requiring researchers to pre-specify them^44,45^. Random Forest models are generally robust to nonlinearity, heterogeneity, some missingness, and certain kinds of measurement error, making them well suited to complex psychological data^46,47^. They also handle many predictors efficiently and provide built-in measures of variable importance that help identify which predictors matter most^45,48^.

A common concern with Random Forest is interpretability, since the model aggregates many decision trees and is often viewed as a black box^49,45^. SHapley Additive exPlanations (SHAP) values address this challenge by quantifying how much each predictor increases or decreases a given prediction, and by indicating the direction and size of each effect^50,51^. SHAP can summarize global patterns across the sample and also provide case-level explanations, improving transparency and supporting actionable insights from complex models^52,53^. Beyond main effects, SHAP can be extended to compute interaction values, which assign portions of a prediction to the combined influence of two predictors that exceed their individual additive effects. Summarizing SHAP interaction values across participants enables identification of the most significant conditional relationships within the fitted model^50,32^.

### Research objectives

We use Random Forest and SHAP to identify and interpret predictors of flourishing among Black American emerging adults. First, we fit a 1,000-tree Random Forest regression to rank psychosocial, health, and behavioral predictors, allowing for curved relationships and interactions without pre-specification^44,45^. Second, we apply SHAP to explain the relative importance and the direction and magnitude of each predictor’s contribution at both the overall and individual levels^51,50^. Third, we investigate whether active coping alters the relationship between discrimination and flourishing, employing SHAP interaction analyses to identify conditional effects in a data-driven manner^54,55^. This combined approach aims to enhance methodological rigor, generate precise and actionable insights, and inform interventions that support the well-being of historically underserved populations.

## Methods

### Participants

We recruited 513 U.S. young adults aged 18 to 29 years who self-identified as Black or African American. The mean age was 23.71 years (SD = 3.36; range = 18 to 29). The sample was 66% women, 32% men, and 1.6% non-binary, transgender, or another gender identity. Educational attainment among those with non-missing data (*n* = 510) was: high school diploma or less, 45.9%; some college without a degree, 23.1%; a 2-year associate, trade, or technical degree or a 4-year bachelor’s degree, 25.9%; some graduate school, 1.4%; and a master’s or doctoral degree, 3.7%. Most participants were never married at the time of the survey (86%); 12% were married, and 2.6% were separated, widowed, or divorced.

### Procedures

Participants were recruited through Dynata, an online research panel. Participants were identified through a targeted recruitment strategy facilitated by Dynata, which applied specific parameters to its participant database to ensure all individuals invited to the Qualtrics survey met defined inclusion criteria. These parameters strictly required participants to identify as Black or African American, to be between the ages of 18 and 29, and to be currently residing in the United States. Dynata sent email or SMS invitations with a secure link to a Qualtrics survey to panel members who had agreed to receive surveys in exchange for panel incentives such as points or sweepstakes. Eligibility was confirmed via a brief screener assessing age (18 to 29 years), U.S. residence, self-identification as Black or African American, and English proficiency. The survey took approximately 20 to 25 min to complete. Participants received Dynata’s standard panel incentive after completion. All study procedures were approved by the university’s Institutional Review Board. All procedures were performed in accordance with relevant guidelines and regulations. Participants provided informed consent electronically prior to participation. No identifying information was collected.

### Measures

#### Flourishing

Flourishing was assessed with the eight-item Flourishing Scale^1^. Items such as “I lead a purposeful and meaningful life” were rated from 0 (strongly disagree) to 6 (strongly agree). We computed the mean of the eight items, with higher scores indicating greater flourishing. Internal consistency in the current sample was high (Cronbach’s α = 0.880).

#### John Henryism active coping (JHAC)

JHAC was measured with the 12-item John Henryism Active Coping Scale^56^. Items such as “When things don’t go the way I want, that makes me work even harder” were rated from 0 (completely false) to 4 (completely true). We averaged the 12 items; higher values reflect greater active coping. Internal consistency in the current sample was high (Cronbach’s α = 0.855).

#### Discrimination

Current discrimination was assessed with four items adapted from the National Epidemiologic Survey on Alcohol and Related Conditions (NESARC). Items asked about the frequency of discriminatory experiences in the past 12 months in settings such as employment, education, housing, courts, or by the police (0 = never to 4 = very often). Lifetime discrimination used a parallel four-item scale asking about experiences across the lifespan (0 to 4). For each scale, we computed the mean of the items; higher scores indicate more frequent discrimination. Current discrimination and lifetime discrimination were treated as two separate predictors in the model. Internal consistency was high for current discrimination (α = 0.837) and lifetime discrimination (α = 0.842).

#### Mental health and physical health

Healthy physical and mental health days were measured using the Centers for Disease Control and Prevention (CDC) Healthy Days health-related quality of life measures^57^. Participants reported the number of days in the past 30 that their physical health was not good (0–30) and the number of days their mental health was not good (0–30). We then reverse-coded these items to create Good Physical Health Days (30 minus physically unhealthy days) and Good Mental Health Days (30 minus mentally unhealthy days).

#### Substance use

Participants reported past-month substance use frequency using items from the Monitoring the Future study. Participants reported past-month frequency of alcohol use (“How often have you gotten drunk?”), cannabis use (“How often have you used marijuana/weed/pot?”), tobacco use (“How often have you used cigarettes, e-cigarettes, or other tobacco products such as cigars or hookah?”), and other drug use (“How often have you used drugs other than marijuana, such as street drugs, psychedelic drugs, or pills, with the intention of getting high?”^58^ All items were measured on a 6-point scale (1 = never to 6 = more than 20 times). Tobacco use was computed as the average frequency of cigarettes, e-cigarettes, and other tobacco products. Internal consistency across tobacco indicators was acceptable (α = 0.725). All four substance-use variables (alcohol, cannabis, tobacco, and other drugs) were entered as separate predictors in the Random Forest.

#### Sleep

Sleep was assessed with two items: inadequate sleep (“How many days in the past 30 did you not get enough rest or sleep?”; 0 to 30 days) and average sleep duration (“On average, how many hours of sleep do you get in a 24-hour period?”; 0 to 24 h).

#### Demographic covariates

Participants self-reported age, gender, education, marital status, employment, parental status, and religion. Gender was represented with a female indicator coded one for women and zero for all other categories. Marital status was coded as a dummy variable with a value of 1 for married and 0 for not married. Employment was dummy-coded with a value of 1 for full-time or part-time employment and 0 for all other statuses. Parental status was dummy-coded with one for a full-time residential parent and zero for not a parent or for non-residential or part-time parenting. Education was recoded and categorized into five levels: high school diploma or less, some college without a degree, associate’s degree, bachelor’s degree, some graduate school, and master’s or doctoral degree. Religion was recoded into five groups: Christian, Jewish, Muslim, other religion, and no religion or unaffiliated. Models included separate indicators for Christian, Jewish, Muslim, and other religion, with no religion or unaffiliated as the reference category.

### Data analysis strategy

#### Data preprocessing

The analytic sample included 513 participants with complete flourishing data. We modeled eleven focal predictors: JHAC, Good Mental Health Days, Good Physical Health Days, current discrimination, lifetime discrimination, alcohol use frequency, tobacco use frequency, cannabis use frequency, other drug use frequency, days without enough rest or sleep, and average hours of sleep per twenty-four hours. In addition, the models included demographic covariates: age, education, religion, employment status, gender, parental status, and having children. Skipped or nonresponse items were treated as missing prior to imputation and model fitting. And sleep values outside their allowable ranges were set to missing. Average sleep duration was winsorized at ± 3 standard deviations to reduce the influence of extreme values while retaining all observations. To prevent information leakage, we imputed missing predictor values separately within the training and test sets using the missForest algorithm with one hundred trees and ten iterations. Because missForest creates only one completed dataset for each data split, it does not capture imputation uncertainty the way multiple imputation does. We report the percentage of missing data for each variable in the Supplement.

#### Training and test split and internal validation

We randomly split the dataset into training (80%, *n* = 410) and test (20%, *n* = 103) sets using a fixed seed (123). Imputation was performed separately within each split using missForest with the same settings. To prevent information leakage, all data-handling steps that could pass information between the training and test sets, including imputation, were carried out separately within each split. After applying range checks and recoding invalid values to missing, the predictors were analyzed on their original scales. Within the training set, we used the Random Forest out-of-bag procedure for internal validation. For each tree, about one-third of cases are left out of its bootstrap sample, and the model predicts those cases. Aggregating these out-of-bag predictions yields unbiased estimates of mean squared error (MSE) and the proportion of variance explained (R^2^), which reduces overfitting and removes the need for separate cross-validation.

#### Random forest modeling and SHAP explanations

We fit a Random Forest regression using the *ranger* package in RStudio. Within the training set, we selected hyperparameters using a training-only grid search optimized on OOB error. The best-performing settings were mtry = 6 and min.node.size = 5, with 1,000 trees. We computed permutation-based variable importance, which measures the drop in model accuracy when a predictor’s values are randomly shuffled. For reproducibility, we used a fixed random seed (123) for the train/test split, model fitting, and SHAP estimation, and a separate seed (456) for the test-set imputation procedure. To improve interpretability, we computed SHapley Additive exPlanation (SHAP) values, which quantify how much each predictor increases or decreases each individual’s predicted flourishing score. SHAP values were calculated using the fastshap package; we evaluated stability across nsim = 50, 200, and 500 and observed highly stable mean absolute SHAP rankings (Spearman ρ = 0.993 for 50 vs. 200; ρ = 0.998 for 200 vs. 500). To examine conditional effects, we also computed SHAP interaction values using the TreeSHAP algorithm implemented in the treeshap package for ranger models. SHAP interaction values break each prediction into its main effects and the added contribution from pairs of predictors, which helps identify when a predictor’s influence depends on the level of another predictor. We summarized interaction strength using the mean absolute interaction contribution across participants and highlighted the strongest interactions, with particular attention to the active coping–discrimination pattern.

## Results

### Random forest model performance

Table 1 presents descriptive statistics for the outcome and all predictors, including means and standard deviations for continuous variables and frequencies for categorical variables. Bivariate correlations among continuous predictors and flourishing are provided in Supplementary Table S1. We trained a 1,000-tree Random Forest on 410 participants and evaluated performance on an independent test set of 103 participants. Using OOB predictions for internal validation, the tuned model explained 26.7% of the variance in flourishing (OOB R^2^ = 0.267). Performance was similar on the test set (R^2^ = 0.245; RMSE = 1.044), indicating good generalization with minimal overfitting. A sensitivity analysis excluding age produced substantively similar performance (OOB R^2^ = 0.267; test R^2^ = 0.244; RMSE = 1.045).

**Table 1:** Descriptive statistics for outcome and predictors.

Variable	Mean	SD	Min–Max
Flourishing	4.20	1.12	0–6
John henryism active coping	2.88	0.66	0.17–4
Good mental health days	17.70	8.53	0–29
Good physical health days	22.00	7.00	0.8–29
Current discrimination	1.34	1.01	0–4
Lifetime discrimination	2.62	0.97	1–5
Alcohol intoxication frequency	1.10	1.35	0–5
Cannabis use frequency	1.60	1.98	0–5
Other drug use frequency	0.37	0.99	0–5
Days without enough rest/sleep	12.70	8.17	1–30
Average sleep duration (hours)	8.12	3.95	1–20
Age	23.70	3.36	18–29

Variable	Category	*n*	%
Tobacco user	Yes	209	41.97
	No	289	58.03
Education	HS diploma or less	26	5.14
	Some college, no degree	207	40.91
	Associate or Bachelor’s	176	34.78
	Some graduate school	71	14.03
	Master’s or Doctoral	26	5.14
Gender	Female	332	65.23
	Male	177	34.77
Married	Yes	59	11.59
	No	450	88.41
Employed	Yes	307	63.69
	No	175	36.31
Full-time residential parent	Yes	129	25.75
	No	372	74.25
Has children	Yes	150	29.88
	No	352	70.12
Religious affiliation	Christian	283	56.60
	Non-religious	157	31.40
	Muslim	18	3.60
	Other	42	8.40

Means and standard deviations are shown for continuous variables.

Percentages are calculated among participants with non-missing data for each variable.

### Variable importance

We estimated each predictor’s importance using the mean absolute SHAP value, which summarizes the average magnitude of each feature’s contribution to individual flourishing predictions (in the model’s prediction units). JHAC was the dominant predictor (mean |SHAP| = 0.314), roughly three times larger than the next predictor, good mental health days (0.122). Several predictors showed smaller but nonzero contributions: days without enough rest (0.049), lifetime discrimination (0.042), education grouped into five levels (0.038), good physical health days (0.037), alcohol use frequency (0.035), cannabis use frequency (0.034), age (0.034), and current discrimination (0.025). Additional effects were minor: having children (0.019), female indicator (0.016), other drug use frequency (0.015), full time residential parent (0.011), and employed (0.010). The smallest importances were tobacco use status (0.009), other religion (0.008), being married (0.008), Muslim indicator (0.007), and the Christian and Jewish indicators (0.003 each). The steep drop from JHAC to all other predictors highlights its central role in the model. Because several predictors are correlated, SHAP attributions can be distributed across related features, so these values should be interpreted as relative contributions within the full model rather than as standalone causal effects^59^.

### SHAP analysis results

**Fig. 1:**
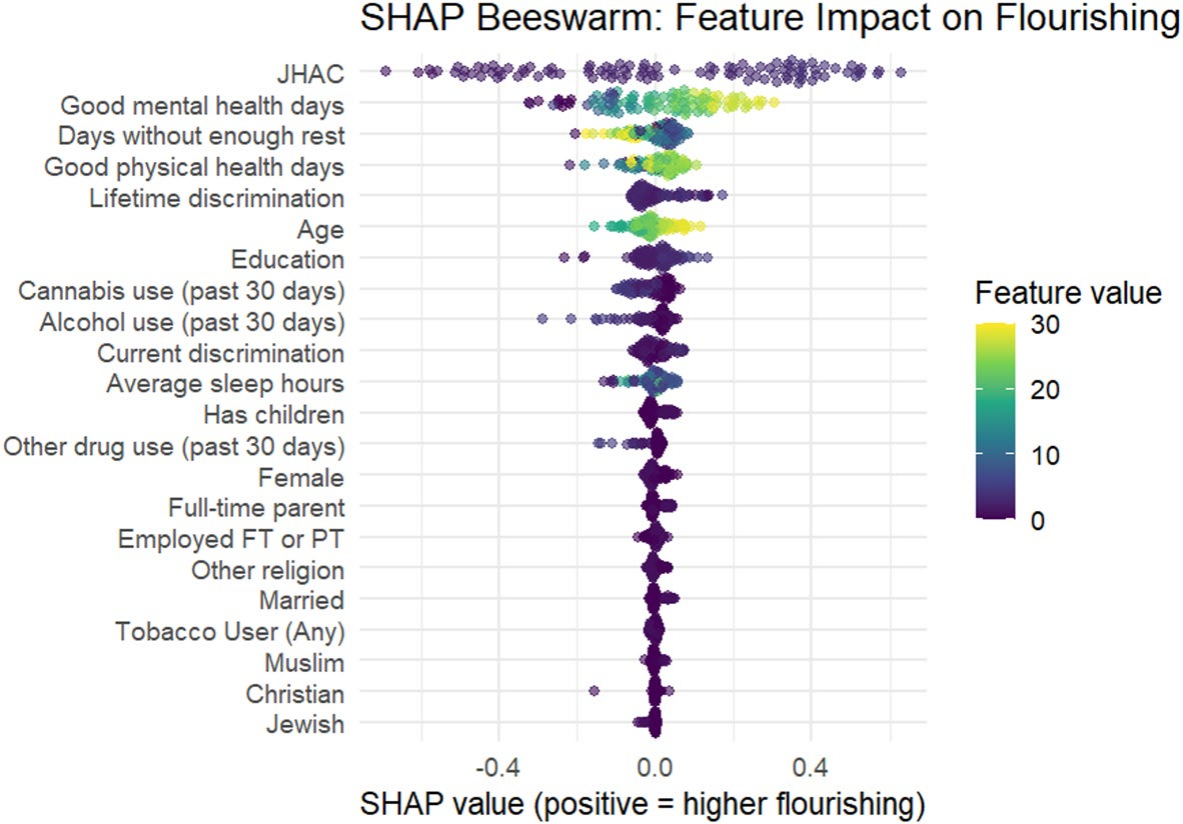
Global SHAP summary (beeswarm) for predictors of flourishing.

The SHAP summary plot (Fig. 1) complements permutation importance by showing how each feature shifts individual predictions in both direction and magnitude relative to the model’s baseline. Consistent with the updated importance rankings, JHAC has by far the widest spread of SHAP values, indicating the strongest influence on predicted flourishing across participants. Higher JHAC values generally align with positive SHAP values (pushing predictions upward), whereas lower JHAC values align with negative SHAP values (pushing predictions downward). Good mental health days shows the next largest spread with a similar pattern, followed by good physical health days, which contributes positively but with a narrower dispersion.

Sleep indicators show opposing patterns: higher values of days without enough rest tend to be associated with negative SHAP values, while higher average sleep hours tends to have a smaller positive contribution. Cannabis use shows relatively small contributions overall, with some individuals exhibiting negative shifts when use is present. Alcohol use and other drug use show slightly higher importance than tobacco use status, although all substance use predictors remain low in contribution relative to the core health variables.

Demographic covariates generally show minor contributions. Education and age exhibit modest, mixed shifts across individuals. Religious indicators contribute little on average, with most SHAP values concentrated near zero. Parent status, marital status, employment, and sex are similarly near zero for most cases. For these binary or one hot coded variables, the color gradient is less informative, so interpretation relies primarily on the horizontal position and spread of points rather than color differences.

Lifetime discrimination ranks higher in importance than current discrimination, although both show smaller but nonzero contributions after accounting for JHAC and mental health. In the summary plot, higher discrimination values tend to align with slightly negative SHAP values for many participants, suggesting a modest downward pull on predicted flourishing predictions. Overall, SHAP results align with permutation importance: JHAC and good mental health days are the primary contributors to the model’s predictions, physical health and sleep add smaller signals, discrimination contributes a modest negative shift, and substance use and demographic variables contribute least within the full model.

### Combined effects

TreeSHAP interaction values indicated conditional effects involving JHAC, including the strongest interaction between good mental health days and JHAC (mean absolute interaction SHAP = 0.0186; Supplementary Table S4). Although current discrimination showed only moderate global importance (Mean |SHAP| = 0.025; Supplementary Table S3), the TreeSHAP interaction results indicated that its contribution to predicted flourishing varied depending on JHAC levels (mean absolute interaction SHAP = 0.0083). These interaction values are model-based attribution metrics rather than regression simple slopes and quantify the extent to which pairs of predictors jointly contribute to the random forest’s predictions beyond their individual main effects.

**Fig. 2:**
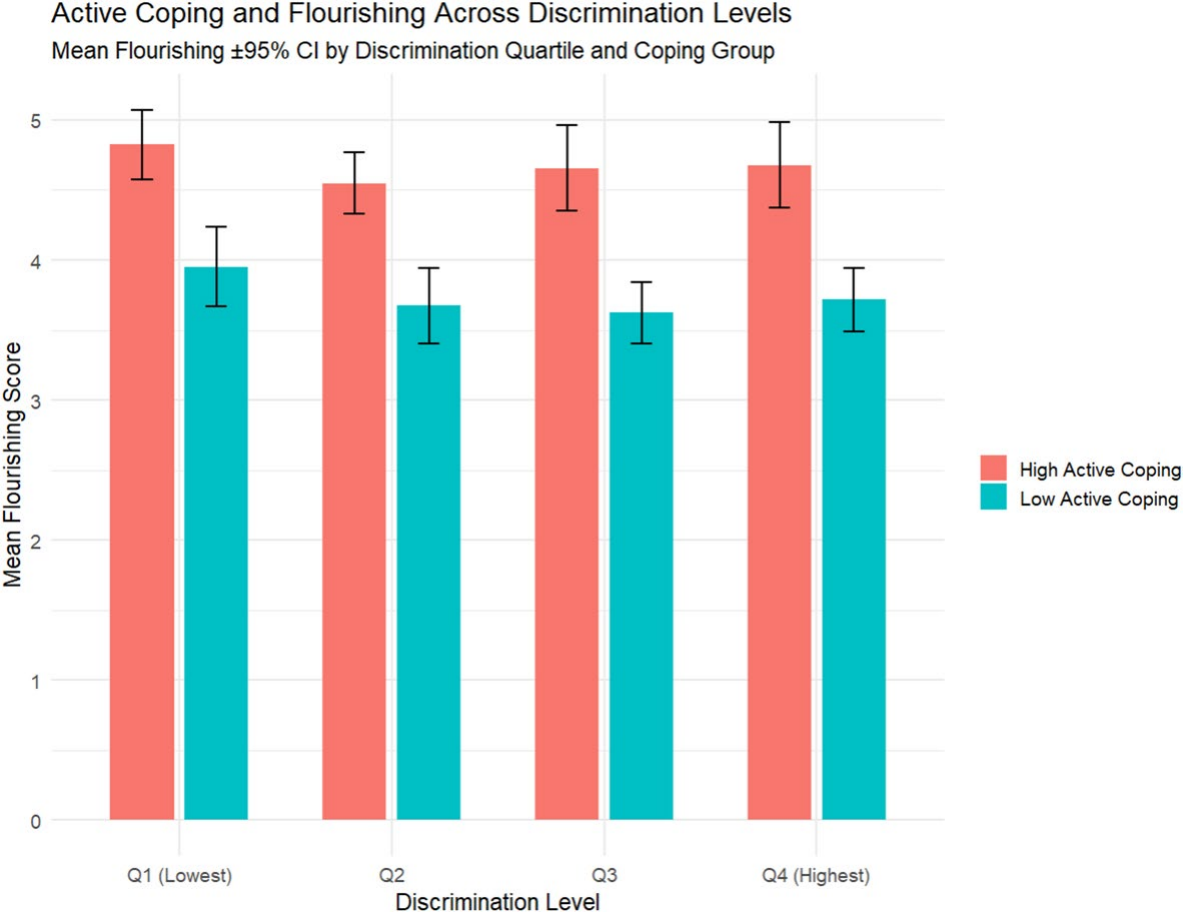
Conditional association of discrimination with flourishing by coping level.

To visually summarize the joint pattern of discrimination and active coping, we created sample based discrimination quartiles and defined lower and higher JHAC as values at or below the 25th percentile and at or above the 75th percentile of the study sample distribution. Figure 2 shows mean flourishing and 95% confidence intervals across discrimination quartiles for the lower JHAC ( < = 25th) and higher JHAC ( > = 75th) groups. Across discrimination levels, flourishing was consistently higher among participants with higher JHAC compared to those with lower JHAC.

Together, these findings suggest two roles for JHAC. First, higher JHAC is associated with higher flourishing on average. Second, JHAC appears to buffer the negative association between current discrimination and flourishing. Individuals with higher JHAC maintain well-being as discrimination increases, while those with lower JHAC show progressively lower flourishing at higher levels of discrimination. Taken together, the findings invite a contextual reading of JHAC as salient on average and as a potential buffer under higher discrimination, interpreted alongside known physiological costs and structural constraints.

## Discussion

John Henryism active coping (JHAC) was the strongest predictor of flourishing in this sample of Black American young adults. Good mental health days contributed next, while good physical health days, discrimination, and the two sleep indicators showed smaller but nonzero unique variance; substance use variables clustered near zero on average. These patterns appeared in both permutation importance and SHAP explanations and remained after adding demographic covariates (age, education, religion, marital status, employment, gender, parental status, and having children), which were small on average in both frameworks. JHAC showed a wide spread of positive SHAP values, whereas many behavioral, sleep, and demographic variables clustered near zero. SHAP interaction values further indicated a buffering pattern: the adverse association between discrimination and flourishing was strongest at low JHAC and diminished as JHAC increased.

JHAC’s prominence in our results aligns with research that frames it as a coping pattern shaped by chronic, racism-related stressors and constrained resources. This literature links JHAC to specific mental health benefits while also documenting the physiological costs of sustained stress^14^ and recognizing the impact of high stress and limited resources on well-being^60^. Importantly, our findings build on existing literature by demonstrating the detrimental effect of discrimination on the flourishing of Black American emerging adults. John Henryism Active Coping (JHAC) may serve as a key psychological resource that helps preserve positive affect in the face of discrimination, thereby promoting flourishing.

In our study, participants with high levels of JHAC maintained consistently high levels of flourishing regardless of discrimination exposure. In contrast, those with low JHAC experienced a steady decline in flourishing as discrimination increased. This buffering effect aligns with resilience and stress-buffering frameworks, which posit that coping resources and culturally grounded strengths can mitigate the psychological consequences of racial discrimination^61^. When young people lack a sense of control over their lives and circumstances, they may be more vulnerable to psychological distress^73^. By engaging in JHAC in response to racism, Black American emerging adults may reclaim a sense of confidence and control that discrimination threatens to erode. John Henryism is theorized as a high-effort coping style that may be psychologically adaptive in some contexts while carrying physiological or long-term costs under chronic stress and constrained resources^15,16,62,63^. Accordingly, we interpret the strong predictive association with flourishing as a pattern of positive functioning in this sample rather than as evidence that high-effort coping is uniformly beneficial across contexts.

Methodologically, combining Random Forest with SHAP explanations allowed us to detect nonlinearities and interactions without prespecifying functional forms and to obtain both global rankings and individual-level attributions that reveal heterogeneity in effects^64^. This approach is well-suited to psychological data in which predictors can interact and linear models are vulnerable to misspecification^44,45^. In our case, it provided stable global importance estimates, identified JHAC as the strongest predictor even after adding sleep, substance use, and demographic covariates, and revealed a JHAC by discrimination buffering pattern that a main-effects linear model could overlook.

Several limitations warrant mention. First, the cross-sectional design prevents causal inference; longitudinal or experimental studies are needed to test directionality^65^. Second, the online panel sample is efficient, but it is a non-probability sample, which can limit generalizability. Representativeness and data quality concerns for commercial panels should be considered^66^. Third, several predictors relied on retrospective self-reports, including discrimination, substance use, and healthy days. These can be affected by recall bias, and using only self-reports can inflate associations. Future studies should combine ecological momentary assessment, administrative records, and biomarkers to triangulate findings^67^. The held-out test set included 103 participants, which reduces the precision of the performance estimates and increases uncertainty around small differences in variable-importance rankings. We therefore interpret the performance and SHAP results as descriptive indicators of the model’s predictive behavior rather than as inferential effect sizes.

Recall windows differed across instruments: health and substance use indicators reflected the past 30 days, whereas discrimination indicators reflected past-year and lifetime exposure. These differences reflect standard time frames embedded in the source measures and are noted as a limitation when interpreting relative predictor importance. Finally, SHAP improves interpretability but remains descriptive and does not establish causality. Combining predictive machine learning with causal inference methods, such as causal forests or targeted maximum likelihood estimation, is a promising direction to move from prediction to causal explanation^68,69^. Future research should prioritize longitudinal replication in representative cohorts to test the stability and direction of the coping effect, mechanistic studies of mediators such as perceived control and social support, and pragmatic trials of culturally adapted coping interventions that assess both psychosocial and physical health outcomes^70^.

## Conclusion

The present study advances the literature by using interpretable machine learning (Random Forest with SHAP) to rank co-occurring psychosocial, health, sleep, substance-use, and demographic predictors of flourishing in Black American emerging adults and to examine conditional effects without prespecifying interactions. Specifically, the current study found that JHAC was the strongest predictor of flourishing; good mental health days contributed next; good physical health days and sleep showed more minor effects (more hours positive, more days without enough rest negative); most substance-use and demographic covariates had minimal unique influence; and higher JHAC appeared to buffer the negative association between current discrimination and flourishing. Nonetheless, the authors urge caution in interpreting JHAC as universally beneficial for emerging adults’ well-being, or in assuming that the modest unique contribution of discrimination in the complete model implies its insignificance. While JHAC may offer psychological advantages for some individuals navigating discrimination, this association warrants careful consideration before being translated into practice.

Although John Henryism has the potential to foster flourishing and serve as a source of resilience, empowerment, and well-being among Black American emerging adults, its physiological costs, particularly when paired with high stress and limited resources, are substantial and well-documented^15^. Implications for practice include prioritizing culturally attuned coping and resilience programs that build problem-solving, mastery, and adaptive persistence, while monitoring potential physiological costs noted in the John Henryism literature^15^. Interventions should pair psychosocial skill building with health monitoring and structural supports^16^. Community-based, peer, and faith-aligned delivery models may be especially acceptable and effective for Black American young adults, given evidence that family, communal, and religious supports are key promotive resources^71^. At the policy level, strengthening individual resources is necessary but not sufficient. Efforts to reduce discrimination and economic disadvantage remain essential because coping resources cannot fully replace reductions in structural stressors^72^.

## Supplementary Information

Below is the link to the electronic supplementary material.

Supplementary Material 1

## Data Availability

Data are not available for sharing. In accordance with the study’s IRB-approved protocol, data will not be banked for future use, and findings are only reported in aggregate. The analysis code used for this study is publicly available on figshare at: kebbeh, Nema; Jelsma, Elizabeth (2026). Predictive Factors of Black Emerging Adults’ Psychological Flourishing. figshare. Software. 10.6084/m9.figshare.31830190.

## References

[1] Diener, E. et al. New measures of well-being. In *The collected works of Ed Diener* (ed Diener, E.) 247–266 (Springer, 2009).

[2] Huppert, F. A. & Whittington, J. E. Psychological well-being and psychological distress: Is it necessary to measure both? *Psychol. Well Being Theory Res. Pract.*. 10.1186/2211-1522-2-3 (2012).

[3] Keyes, C. L. M. The mental health continuum: From languishing to flourishing in life. *J. Health Soc. Behav.* **43**, 207–222 (2002). 12096700

[4] Mjøsund, N. H. A. Salutogenic mental health model: Flourishing as a metaphor for good mental health. In *Health Promotion in Health Care – Vital Theories and Research* (eds Haugan, G. & Eriksson, M.) 47–59 (Springer, (2021). 36315728

[5] Arnett, J. J. Emerging adulthood: A theory of development from the late teens through the twenties. *Am. Psychol.* **55**, 469–480 (2000). 10842426

[6] Wood, D. L. et al. *Handbook of Life Course Health Development* (eds Halfon, N., Forrest, C. B., Lerner, R. M. & Faustman, E. M.)123–143 (Springer, 2018). 31314220

[7] Solmi, M. et al. Age at onset of mental disorders worldwide: Large-scale meta-analysis of 192 epidemiological studies. *Mol. Psychiatry* **27**, 281–295 (2022). 34079068 10.1038/s41380-021-01161-7 PMC8960395

[8] Kelly-Hedrick, M., Rodriguez, M. M., Ruble, A. E., Wright, S. M. & Chisolm, M. S. Measuring flourishing among internal medicine and psychiatry residents. *J. Grad. Med. Educ.* **12**, 312–319 (2020). 32595851 10.4300/JGME-D-19-00793.1 PMC7301920

[9] Volstad, C. et al. You have to be okay with okay: Experiences of flourishing among university students transitioning directly from high school. *Int. J. Qual. Stud. Health Well Being* **15**, 1834259 (2020). 33106113 10.1080/17482631.2020.1834259 PMC7594843

[10] Collins-Anderson, A., Vahedi, L., Hutson, W. & Hudson, D. Intersectionality and mental health among emerging adult Black American men: A scoping review. *Curr. Psychiatry Rep.* **24**, 819–830 (2022). 36449172 10.1007/s11920-022-01386-5 PMC9994382

[11] Williams, D. R. Stress and the mental health of populations of color: Advancing our understanding of race-related stressors. *J. Health Soc. Behav.* **59**, 466–485 (2018). 30484715 10.1177/0022146518814251 PMC6532404

[12] García Coll, C. et al. V. An integrative model for the study of developmental competencies in minority children. *Child Dev.* **67**(5), 1891–1914. 10.2307/1131600 (1996). 9022222

[13] Hope, E. C., Hoggard, L. S. & Thomas, A. Emerging into adulthood in the face of racial discrimination: Physiological, psychological, and sociopolitical consequences for african american youth. *Transl Issues Psychol. Sci.* **1**, 342–351 (2015).

[14] Bronder, E. C., Speight, S. L., Witherspoon, K. M. & Thomas, A. J. John Henryism, depression, and perceived social support in Black women. *J. Black Psychol.* **40**(2), 115–137. 10.1177/0095798412474466 (2014).

[15] Bennett, G. G. et al. Stress, coping, and health outcomes among African-Americans: A review of the John Henryism hypothesis. *Psychol. Health* **19**(3), 369–383. 10.1080/0887044042000193505 (2004).

[16] James, S. John Henryism and the health of African-Americans. *Cult. Med. Psychiatry* **18**(2), 163–182. 10.1007/BF01379448 (1994). 7924399 10.1007/BF01379448

[17] Volpe, V. V., Rahal, D., Holmes, M. & Rivera, S. Z. Is hard work and high effort always healthy for Black college students? John Henryism in the face of racial discrimination. *Emerg. Adulthood* **8**(3), 245–252 (2020).

[18] Bernard, D. L., Jones, S. C. T. & Volpe, V. V. Impostor phenomenon and psychological well-being: The moderating roles of John Henryism and school racial composition among Black college students. *J. Black Psychol.*10.1177/0095798420924529 (2020). 10.1177/0095798420924529 PMC7377315 32704193

[19] Haritatos, J., Mahalingam, R. & James, S. A. John Henryism, self-reported physical health indicators, and the mediating role of perceived stress among high socio-economic status Asian immigrants. *Soc. Sci. Med.* **64**(6), 1192–1203 (2007). 17174456 10.1016/j.socscimed.2006.10.037

[20] Hill, L. K. & Hoggard, L. S. Active coping moderates associations among race-related stress, rumination, and depressive symptoms in emerging adult African American women. *Dev. Psychopathol.* **30**, 1817–1835 (2018). 30451137 10.1017/S0954579418001268 PMC6839900

[21] Jelsma, E., Chen, S. & Varner, F. Working harder than others to prove yourself: High-effort coping as a buffer between teacher-perpetrated racial discrimination and mental health among Black American adolescents. *J. Youth Adolesc.* **51**(4), 694–707 (2022). 35094198 10.1007/s10964-021-01563-4 PMC8930523

[22] Matthews, D. D., Hammond, W. P., Nuru-Jeter, A., Cole-Lewis, Y. & Melvin, T. Racial discrimination and depressive symptoms among African-American men: The mediating and moderating roles of masculine self-reliance and John Henryism. *Psychol. Men Masculinity* **14**(1), 35–46. 10.1037/a0028436 (2013). 10.1037/a0028436 PMC6197817 30364828

[23] Lo-oh, J. L. From positive psychology to positive development: Overcoming adversity and flourishing in emerging adulthood. *J. Cult. Soc. Dev.* **46**, 39 (2019).

[24] Arnett, J. J. Identity development from adolescence to emerging adulthood: What we know and (especially) don’t know. In *The Oxford Handbook of Identity Development* 53–64 (2015).

[25] De la Fuente, R., Parra, A. & Sánchez Queija, I. & Lizaso Elgarresta, I. Flourishing during emerging adulthood from a gender perspective. *J. Happiness Stud.* **21** (2019).

[26] Nelson, L. J. & Padilla-Walker, L. M. Flourishing and floundering in emerging adult college students. *Emerg. Adulthood* **1**, 67–78 (2013).

[27] Vance, T. Addressing Mental Health in the Black Community. Columbia University Department of Psychiatry. https://www.columbiapsychiatry.org/news/addressing-mental-health-black-community (2019).

[28] Wickham, S. R., Amarasekara, N. A., Bartonicek, A. & Conner, T. S. The big three health behaviors and mental health and well-being among young adults: A cross-sectional investigation of sleep, exercise, and diet. *Front. Psychol.* **11**, 579205 (2020). 33362643 10.3389/fpsyg.2020.579205 PMC7758199

[29] Patterson, A., Voichoski, E., Fucinari, J. & Smith, V. *Flourishing Bolstering the Mental Health of Students at HBCUs and PBIs Research Findings, Discussion & Recommendations* (United Negro College Fund, 2025).

[30] Sofija, E., Harris, N., Sebar, B. & Phung, D. Who are the flourishing emerging adults on the urban East Coast of Australia? *Int. J. Environ. Res. Public. Health* **18**, 1125 (2021). 33514003 10.3390/ijerph18031125 PMC7908618

[31] Kang, Y., Kim, J. & Kim, Y. Contributions of physical activity and positive psychological functioning to flow and well-being. *J. Happiness Stud.* **24**(5), 1451–1465. 10.1007/s10902-023-00585-0 (2023).

[32] Kim, J., Park, S. & Lee, S. Better sleep quality and higher physical activity levels predict lower emotion dysregulation among persons with major depressive disorder. *BMC Psychol.* **11**, 213. 10.1186/s40359-023-01213-3 (2023). 37226277 10.1186/s40359-023-01213-3 PMC10207795

[33] Gerrard, M. et al. Coping with racial discrimination: The role of substance use. *Psychol. Addict. Behav.* **26**, 550–560 (2012). 22545585 10.1037/a0027711 PMC4079542

[34] Jones, D. M., Masyn, K. E. & Spears, C. A. Associations among discrimination, psychological functioning, and substance use among US Black adults aged 18–28: Moderation by racial attribution and sex. *J. Subst. Use Addict. Treat.* **153**, 209080 (2023). 37230392 10.1016/j.josat.2023.209080 PMC10526892

[35] Ng, V. K. Y. & Cribbie, R. A. Using the gamma generalized linear model for modeling continuous, skewed and heteroscedastic outcomes in psychology. *Curr. Psychol.* **36**, 225–235 (2017).

[36] Rimpler, A., Kiers, H. A. L. & van Ravenzwaaij, D. To interact or not to interact: The pros and cons of including interactions in linear regression models. *Behav. Res. Methods* **57**, 92 (2025). 39920441 10.3758/s13428-025-02613-6 PMC11805792

[37] Ernst, A. F. & Albers, C. J. Regression assumptions in clinical psychology research practice—a systematic review of common misconceptions. *PeerJ* **5**, e3323 (2017). 28533971 10.7717/peerj.3323 PMC5436580

[38] Field, A. P. & Wilcox, R. R. Robust statistical methods: A primer for clinical psychology and experimental psychopathology researchers. *Behav. Res. Ther.* **98**, 19–38 (2017). 28577757 10.1016/j.brat.2017.05.013

[39] Finsaas, M. C. & Goldstein, B. L. Do simple slopes follow-up tests lead us astray? Advancements in the visualization and reporting of interactions. *Psychol. Methods* **26**, 38–60 (2021). 32309961 10.1037/met0000266

[40] Baranger, D. A. A. et al. Tutorial: Power analyses for interaction effects in cross-sectional regressions. *Adv. Methods Pract. Psychol. Sci.* **6**, 25152459231187531 (2023). 10.1177/25152459231187531 PMC12341451 40799847

[41] Cheung, G. W. & Lau, R. S. Accuracy of parameter estimates and confidence intervals in moderated mediation models: A comparison of regression and latent moderated structural equations. *Organ. Res. Methods* **20**, 746–769 (2017).

[42] Kiefer, C., Wilker, S. & Mayer, A. Interactions between latent variables in count regression models. *Behav. Res. Methods* **56**, 8932–8954 (2024). 39187739 10.3758/s13428-024-02483-4 PMC11525413

[43] Siemsen, E., Roth, A. & Oliveira, P. Common method bias in regression models with linear, quadratic, and interaction effects. *Organ. Res. Methods* **13**, 456–476 (2010).

[44] Genuer, R., Poggi, J. M. & Tuleau-Malot, C. VSURF: An R package for variable selection using random forests. *R J.* **7**, 19–33 (2015).

[45] Denisko, D. & Hoffman, M. M. Classification and interaction in random forests. *Proc. Natl. Acad. Sci. U. S. A.* **115**, 1690–1692 (2018). 10.1073/pnas.1800256115 PMC5828645 29440440

[46] Tang, F. & Ishwaran, H. Random forest missing data algorithms. *Stat. Anal. Data Min.* **10**, 363–377 (2017). 10.1002/sam.11348 PMC5796790 29403567

[47] Zhu, T. Analysis on the applicability of the random forest. In *Journal of Physics: Conference Series* Vol. 1607, 012123 (2020).

[48] Ghosh, D. & Cabrera, J. Enriched random forest for high dimensional genomic data. *IEEE ACM Trans. Comput. Biol. Bioinform.* **19**, 2817–2828 (2021). 10.1109/TCBB.2021.3089417 PMC9923687 34129502

[49] Scornet, E., Biau, G. & Vert, J. P. Consistency of random forests. *Ann. Stat.* **43**, 1716–1741 (2015).

[50] Lundberg, S. & Lee, S. I. A Unified approach to interpreting model predictions. https://arxiv.org/abs/1705.07874 (2017).

[51] Rodríguez-Pérez, R. & Bajorath, J. Interpretation of machine learning models using shapley values: Application to compound potency and multi-target activity predictions. *J. Comput. Aided Mol. Des.* **34**, 1013–1026 (2020). 32361862 10.1007/s10822-020-00314-0 PMC7449951

[52] Padarian, J., McBratney, A. B. & Minasny, B. Game theory interpretation of digital soil mapping convolutional neural networks. *Soil* **6**, 389–397 (2020).

[53] Hu, C. et al. Interpretable machine learning for early prediction of prognosis in sepsis: A discovery and validation study. *Infect. Dis. Ther.* **11**, 1117–1132 (2022). 35399146 10.1007/s40121-022-00628-6 PMC9124279

[54] Eker, R. & Aydın, A. Predicting potential fire severity in Türkiye’s diverse forested areas: A SHAP-integrated random forest classification approach. *Stoch. Environ. Res. Risk Assess.* **38**, 4607–4628 (2024).

[55] Ding, X. & Kwon, T. J. Enhancing winter road maintenance with explainable AI: SHAP analysis for interpreting machine learning models in road friction estimation. *Can. J. Civ. Eng.* **51**(5), 407–420 (2024).

[56] James, S. A., Strogatz, D. S., Wing, S. B. & Ramsey, D. L. Socioeconomic status, John Henryism, and hypertension in blacks and whites. *Am. J. Epidemiol.* **126**(4), 664–673 (1987). 3631056 10.1093/oxfordjournals.aje.a114706

[57] Moriarty, D. G., Zack, M. M. & Kobau, R. The centers for disease control and prevention’s healthy days measures—Population tracking of perceived physical and mental health over time. *Health Qual. Life Outcomes* **1**, 37 (2003). 14498988 10.1186/1477-7525-1-37 PMC201011

[58] Johnston, L. D. et al. *Monitoring the Future national survey results on drug use 1975–2019: Key findings on adolescent drug use* (Institute for Social Research, University of Michigan, 2019).

[59] Molnar, C. *Interpretable Machine Learning: A Guide for Making Black Box Models Explainable* 3rd ed. christophm.github.io/interpretable-ml-book/ (2025).

[60] Carothers, K. J., Arizaga, J. A., Carter, J. S., Taylor, J. & Grant, K. E. The costs and benefits of active coping for adolescents residing in urban poverty. *J. Youth Adolesc.* **45**, 1323–1337 (2016). 27138173 10.1007/s10964-016-0487-1

[61] Spence, N. D., Wells, S., Graham, K. & George, J. Racial discrimination, cultural resilience, and stress. *Can. J. Psychiatry* **61**, 298–307 (2016). 27254805 10.1177/0706743716638653 PMC4841285

[62] James, S. A., Hartnett, S. A. & Kalsbeek, W. D. John Henryism and blood pressure differences among Black men. *J. Behav. Med.* **6**(3), 259–278. 10.1007/BF01315113 (1983). 6663614 10.1007/BF01315113

[63] Robinson, M. N. & Thomas Tobin, C. S. Is John Henryism a health risk or resource? Exploring the role of culturally relevant coping for physical and mental health among Black Americans. *J. Health Soc. Behav.* **62**(2), 136–151. 10.1177/00221465211009142 (2021). 34100655 10.1177/00221465211009142 PMC8370445

[64] Haddouchi, M. & Berrado, A. A survey of methods and tools used for interpreting Random Forest. In *2019 1st International Conference on Smart Systems and Data Science (ICSSD)* 1–6 (2019).

[65] Demetrescu, M. & Homm, U. Directed tests of no cross-sectional correlation in large-N panel data models. *J. Appl. Econom.* **31**, 4–31 (2016).

[66] Lehdonvirta, V., Oksanen, A., Räsänen, P. & Blank, G. Social media, web, and panel surveys: using non-probability samples in social and policy research. *Policy Internet* **13**, 134–155 (2021).

[67] Bertz, J. W., Epstein, D. H. & Preston, K. L. Combining ecological momentary assessment with objective, ambulatory measures of behavior and physiology in substance-use research. *Addict. Behav.* **83**, 5–17 (2018). 29174666 10.1016/j.addbeh.2017.11.027 PMC5955807

[68] Wager, S. & Athey, S. Estimation and inference of heterogeneous treatment effects using random forests. *J. Am. Stat. Assoc.* **113**, 1228–1242 (2018).

[69] Van Der Laan, M. J. & Rose, S. *Targeted Learning in Data Science: Causal Inference for Complex Longitudinal Studies* (Springer, 2018).

[70] Eccles, M. P. & Mittman, B. S. Welcome to Implementation Science. *Implement. Sci.* **1**, 1 (2006).

[71] Kherad-Pajouh, S. & Renaud, O. A general permutation approach for analyzing repeated measures ANOVA and mixed-model designs. *Stat. Pap.* **56**, 947–967 (2015).

[72] Barajas, C. B. et al. Coping, discrimination, and physical health conditions among predominantly poor, urban African Americans: Implications for community-level health services. *J. Community Health* **44**, 954–962 (2019). 30915675 10.1007/s10900-019-00650-9 PMC6708452

[73] Keyes, C. L. M. Promoting and protecting mental health as flourishing: A complementary strategy for improving national mental health. *Am. Psychol.* **62**, 95–108 (2007). 17324035 10.1037/0003-066X.62.2.95

