# Supplementary material for: Predictive factors of Black American emerging adults’ psychological flourishing: a random forest analysis
Source: Sci Rep. 2026 Apr 17;16:17868. doi: 10.1038/s41598-026-46864-5 (PMC13250128; doi:10.1038/s41598-026-46864-5)
Supplement: Supplementary file 1 — Supplementary Material 1 [file 41598_2026_46864_MOESM1_ESM.docx]

**Supplementary Table S1**

| Variable | 1 | 2 |  | 3 | 4 | 5 | 6 | 7 | 8 | 9 | 10 | 11 | 12 | 13 | 14 |
| --- | --- | --- | --- | --- | --- | --- | --- | --- | --- | --- | --- | --- | --- | --- | --- |
| 1. Flourishing | — |  |  |  |  |  |  |  |  |  |  |  |  |  |  |
| 2. John Henryism Active Coping | .52** | — |  |  |  |  |  |  |  |  |  |  |  |  |  |
| 3. Good Mental Health Days | .30** | .21** |  | — |  |  |  |  |  |  |  |  |  |  |  |
| 4. Good Physical Health Days | .15** | .11* |  | .33** | — |  |  |  |  |  |  |  |  |  |  |
| 5. Current Discrimination | -0.06 | -.13** |  | -.14** | -.26** | — |  |  |  |  |  |  |  |  |  |
| 6. Lifetime Discrimination | -0.06 | -.13** |  | -.19** | -.20** | .77** | — |  |  |  |  |  |  |  |  |
| 7. Alcohol Use Frequency | -.10* | 0.01 |  | -.13** | -.23** | .34** | .32** | — |  |  |  |  |  |  |  |
| 8. Tobacco Use (Any) | -0.03 | 0.05 |  | -.13** | -0.09 | .24** | .27** | .46** | — |  |  |  |  |  |  |
| 9. Cannabis Use Frequency | -0.08 | 0.02 |  | -.11* | -0.02 | .15** | .20** | .49** | .51** | — |  |  |  |  |  |
| 10. Other Drug Use Frequency | -.11* | -0.09 |  | -.16** | -.28** | .33** | .27** | .45** | .38** | .26** | — |  |  |  |  |
| 11. Days Without Rest or Sleep | -.10* | -0.01 |  | -.38** | -.35** | .15** | .14** | .10* | 0.04 | 0.01 | 0.07 | — |  |  |  |
| 12. Average Sleep Duration | -0.09 | -.15** |  | -.27** | -.50** | .21** | .14** | .13** | .12* | 0.01 | .29** | .21** | — |  |  |
| 13. Age | .12* | 0.09 |  | 0.08 | 0.04 | -0.01 | -0.05 | .13** | .18** | 0.06 | -0.02 | -0.01 | — | — |  |
| 14. Education Level | .16** | 0.07 |  | 0.07 | 0.02 | 0.02 | 0.03 | 0.05 | -0.04 | -0.06 | -0.01 | 0 | .15** | — | — |

Bivariate Correlations Among Study Variables

Note. p < .05. p < .01.

**Supplementary Table S2**

Model performance: baseline, no-age sensitivity, and tuned model

| Model | OOB R² | Test R² | Test RMSE |
| --- | --- | --- | --- |
| Baseline (with age) | 0.250 | 0.227 | 1.057 |
| Sensitivity (no age) | 0.267 | 0.244 | 1.045 |
| Tuned (grid best) | 0.267 | 0.245 | 1.044 |

*Note.* Tuning used a training-only grid search optimized on OOB error; best settings were mtry = 6 and min.node.size = 5 (1,000 trees).

**Supplementary Table S3**

Top predictors by mean absolute SHAP value (nsim = 200)

| Predictor | Mean \|SHAP\| (nsim=200) |
| --- | --- |
| John Henryism Active Coping | 0.314 |
| Good Mental Health Days | 0.122 |
| Days Without Rest or Sleep | 0.049 |
| Lifetime Discrimination | 0.042 |
| Education Level | 0.038 |
| Good Physical Health Days | 0.037 |
| Alcohol Use Frequency | 0.035 |
| Cannabis Use Frequency | 0.034 |
| Age | 0.034 |
| Current Discrimination | 0.025 |

*Note.* Mean absolute SHAP values summarize average magnitude of contribution to predictions on the held-out test set.

**Supplementary Table S4**

Top TreeSHAP Interaction Effects by Mean Absolute Interaction Value

| Feature 1 | Feature 2 | Mean \|interaction\| |
| --- | --- | --- |
| Good Mental Health Days | John Henryism Active Coping | 0.0186 |
| Good Physical Health Days | John Henryism Active Coping | 0.0104 |
| Lifetime Discrimination | Good Mental Health Days | 0.0086 |
| Current Discrimination | John Henryism Active Coping | 0.0083 |
| Average Sleep Duration | John Henryism Active Coping | 0.0076 |
| Current Discrimination | Good Mental Health Days | 0.0067 |
| Days Without Rest or Sleep | John Henryism Active Coping | 0.0067 |
| Days Without Rest or Sleep | Cannabis Use Frequency | 0.0066 |
| Good Physical Health Days | Good Mental Health Days | 0.0064 |
| Cisgender Female | John Henryism Active Coping | 0.0059 |

*Note.* TreeSHAP interaction values represent the mean absolute pairwise contribution of two predictors to the random forest’s predictions beyond their additive main effects, averaged across the held-out test set. Larger values indicate stronger interaction influence on predicted flourishing.

**Supplementary Table S5**

Missing data percentages

| Variable | Percentage missing (%) |
| --- | --- |
| Good physical health days | 28.68% |
| Good mental health days | 15.13% |
| Days without enough rest or sleep | 11.20% |
| John Henryism active coping | 5.30% |
| Employment status | 5.30% |
| Average sleep duration (hours) | 4.91% |
| Lifetime discrimination | 3.14% |
| Current discrimination | 1.77% |
| Tobacco user status | 2.16% |
| Religious preference | 1.77% |
| Full-time residential parent | 1.57% |
| Has children | 1.38% |

Note. Percentages are calculated based on the total sample of 513 participants.
